# The interactive effects of input and output on managing fluid balance in patients with acute kidney injury requiring continuous renal replacement therapy

**DOI:** 10.1186/s13054-019-2633-0

**Published:** 2019-10-29

**Authors:** Jong Hyun Jhee, Hye Ah Lee, Seonmi Kim, Youn Kyung Kee, Ji Eun Lee, Shina Lee, Seung-Jung Kim, Duk-Hee Kang, Kyu Bok Choi, Hyung Jung Oh, Dong-Ryeol Ryu

**Affiliations:** 1 Division of Nephrology, Department of Internal Medicine, Gangnam Severance Hospital, Yonsei University College of Medicine, Seoul, Republic of Korea; 2 Clinical Trial Center, Ewha Womans University Mokdong Hospital, Seoul, Republic of Korea; 3 Department of Internal Medicine, College of Medicine, Ewha Womans University Mokdong Hospital, 1071, Anyangcheon-ro, Yangcheon-gu, Seoul, 07985 Republic of Korea; 4 Department of Internal Medicine, Hangang Sacred Heart Hospital, Hallym University, Seoul, Republic of Korea; 5 Ewha Institute of Convergence Medicine, Ewha Womans University Mokdong Hospital, 1071, Anyangcheon-ro, Yangcheon-gu, Seoul, 07985 Republic of Korea; 6 Research Institute for Human Health Information, Ewha Womans University Mokdong Hospital, Seoul, Republic of Korea

**Keywords:** Cumulative fluid balance, Cumulative input, Cumulative output, All-cause mortality, Acute kidney injury, Continuous renal replacement therapy

## Abstract

**Background:**

The interactive effect of cumulative input and output on achieving optimal fluid balance has not been well elucidated in patients with acute kidney injury (AKI) requiring continuous renal replacement therapy (CRRT). This study evaluated the interrelation of fluid components with mortality in patients with AKI requiring CRRT.

**Methods:**

This is a retrospective observational study conducted with a total of 258 patients who were treated with CRRT due to AKI between 2016 and 2018 in the intensive care unit of Ewha Womans University Mokdong Hospital. The amounts of fluid input and output were assessed at 24-h and 72-h from the initiation of CRRT. The study endpoints were 7- and 28-day all-cause mortality.

**Results:**

The mean patient age was 64.7 ± 15.8 years, and 165 (64.0%) patients were male. During the follow-up, 7- and 28-day mortalities were observed in 120 (46.5%) and 157 (60.9%) cases. The patients were stratified into two groups (28-day survivors vs. non-survivors), and the cumulative fluid balances (CFBs) at 24 h and 72 h were significantly higher in the 28-day non-survivors compared with the survivors. The increase in 24-h and 72-h CFB was significantly associated with an increase in 7- and 28-day mortality risks. To examine the interactive effect of cumulative input or output on the impact of CFB on mortality, we also stratified patients into three groups based on the tertile of 24-h and 72-h cumulative input or output. The increases in 24-h and 72-h CFBs were still significantly related to the increases in 7-day and 28-day mortality, irrespective of the cumulative input. However, we did not find significant associations between increase in 24-h and 72-h CFB and increase in mortality risk in the groups according to cumulative output tertile.

**Conclusions:**

The impact of cumulative fluid balance on mortality might be more dependent on cumulative output. The physicians need to decrease the cumulative fluid balance of CRRT patients as much as possible and consider increasing patient removal.

## Background

Fluid overload has been associated with aggravating renal dysfunction, an increase in length of intensive care unit (ICU) stay, and an elevation in mortality risk [1–4]. Hence, negative fluid balance is regarded as an essential strategy to improve survival rates in critically ill patients, and continuous renal replacement therapy (CRRT) has been widely used for fluid regulation in critically ill patients with acute kidney injury (AKI) [5–7]. However, there is no consensus on the optimal fluid management for such patients [8–12]. Several recent studies revealed that an increase of cumulative fluid balance (CFB) was significantly associated with an increase in mortality risk in critically ill patients [13–17], while Balakumar et al. showed that both positive and negative fluid balance were associated with higher mortality rates compared with even fluid balance [18]. Furthermore, Silversides et al. reported that active removal of fluid using RRT in critically ill patients was associated with less survival benefit compared with standard care [19–21].

Fluid resuscitation for critically ill patients with AKI has both advantages and disadvantages. Fluid resuscitation can help keep patients hemodynamically stable, maintain renal perfusion, and prevent further ischemic injury due to ongoing renal dysfunction. However, accumulating interstitial fluid hinders oxygen delivery to cells from blood vessels and can lead to renal ischemia and multi-organ failure [9, 12, 22]. Thus, optimal management of fluid balance in patients with AKI, especially those requiring CRRT, still needs to be investigated. In addition, most physicians have focused on CFB, even though CFB is determined by the amount of input and output. However, there is still no definite answer for which strategies better benefit survival: does higher output translate into better clinical outcomes, or does lower input translate into better clinical outcomes. Therefore, in this study, we investigated the association between CFB, cumulative input and output amounts at 24-h and 72-h after initiation of CRRT, and mortality risk among patients with AKI receiving CRRT.

## Methods

### Study population

We conducted a retrospective review of patients aged 18 years or older who were treated with CRRT due to AKI between 2016 and 2018 in the ICU of Ewha Womans University Mokdong Hospital. A total 330 patients were initially screened. Exclusion criteria included patients with end-stage renal disease (ESRD) on chronic dialysis or those who underwent kidney transplantation, patients who died within 24-h immediately after CRRT initiation, and patients with missing fluid status data. Finally, a total of 258 patients with 24-h assessment of fluid status and 191 patients with 72-h assessment of fluid status were analyzed (Additional file 1: Figure S1). All the enrolled patients received CRRT management for longer than 24-h. This study was approved by the Institutional Review Board of Ewha Womans University, College of Medicine (IRB No. EUMC 2018-11-019). The need for informed consent from patients was waived because of the retrospective study design. All clinical investigations were conducted in accordance with 2013 Declaration of Helsinki guidelines.

### Data collection

Baseline characteristics of the enrolled patients were collected at the time of CRRT initiation, including age, sex, body mass index (BMI), cause of AKI, systolic blood pressure (SBP), diastolic blood pressure (DBP), mean arterial pressure (MAP), comorbidities (hypertension, diabetes mellitus, cardiovascular disease, malignancy), Charlson Comorbidity Index (CCI), Sequential Organ Failure Assessment (SOFA) score, Acute Physiology and Chronic Health Evaluation (APACHE) II score, and Glasgow Coma Score (GCS). In addition, the needs for mechanical ventilation and fraction of inspired oxygen (FiO2) were investigated, and the prescriptions for CRRT including target clearance, dialysate flow rate, replacement flow rate, and use of anticoagulation were also assessed. Laboratory data were collected at the initiation of CRRT, and estimated glomerular filtration rate (eGFR) was calculated using the Modification of Diet in Renal Disease equation [23]. The definition of AKI, based on the Kidney Disease: Improving Global Outcomes clinical practice guidelines for AKI, was the presence of at least one of the following criteria: (1) an increase in serum creatinine level ≥ 0.3 mg/dL within 48-h, (2) an increase in serum creatinine level to ≥ 1.5 times the baseline level that was known or presumed to have occurred within the previous 7 days, or (3) urine volume < 0.5 mL/kg/h for 6-h [24].

### CRRT protocol

The decision to initiate CRRT and the CRRT settings for target clearance; blood flow, dialysate, and replacement fluid rates; and anticoagulation administration were determined through discussion and consultation with nephrologists [25]. The criteria for CRRT initiation were medically intractable or persistent electrolyte imbalance and/or metabolic acidosis and decreased urine output with volume overload and/or progressive azotemia. Hemodynamic instability was also an important indication. Generally, vascular access for CRRT was via a femoral venous catheter, and the pre-dilution method of continuous venovenous hemodiafiltration was usually performed. After CRRT initiation, attending physicians and experienced nurses monitored body weight, urine output, laboratory results, actual delivered dose, and the hemodynamic status of the patients and discussed the results with nephrologists to maintain CRRT adequacy.

### Fluid status assessment

The amounts of cumulative fluid input and output were assessed for 24-h and 72-h from the initiation of CRRT, and the CFB was calculated as cumulative input − cumulative output. We also investigated the type of infused fluid, including 0.9% sodium chloride, 0.45% sodium chloride, dextrose, plasma solution, lactate Ringer’s solution, 20% albumin, and enteral nutritional fluid, and compared the administered type between the 28-day survivors and non-survivors.

### Statistical analysis

Patients were stratified into two groups: 28-day survivors vs. non-survivors. The baseline characteristics of the groups were compared using the independent *t*-test for continuous variables and the chi-squared test for categorical variables. Continuous variables are presented as the mean ± standard deviation, and categorical variables are presented as numbers and percentages. The non-normally distributed variables were expressed as medians (25th–75th percentiles) and were compared using the Mann-Whitney *U* or Kruskal-Wallis test. The study endpoints were all-cause mortality on the 7- and 28-day time points after commencing CRRT, and Cox proportional hazard analyses were performed to examine the effects of CFB on mortality. Significant covariates were identified by univariable analysis (*P* < 0.05), and the clinically important variables were selected for multivariable analysis. Age, sex, BMI, CCI, medical history, and eGFR at CRRT initiation were adjusted for multivariable Cox proportional regression analyses, as were the MAP, hemoglobin, SOFA score, use of vasopressor, and type of fluid administration at each time point (24-h and 72-h from CRRT start). Before investigating the interactive effect of cumulative input or output on the impact of CFB on mortality, multicollinearity between CFB and cumulative input or output was assessed based on the variance inflation factor (VIF). However, we found that the VIF was too large to adjust the cumulative input or output to show the effect of CFB on mortality risk, so we decided to analyze the effect of cumulative input and output on the impact of CFB on mortality after stratifying the patients. Thus, we stratified patients into three groups based on the tertile of 24-h and 72-h cumulative input or output and investigated the impact of CFB on the mortality in each group. All statistical tests were conducted using a two-tailed 95% confidence interval (CI), and a *P* value of less than 0.05 was considered statistically significant. All descriptive and survival analyses were performed using SPSS for Windows, version 23.0 (IBM, Armonk, NY, USA) and R software version 3.3.1 (R Development Core Team, Vienna, Austria).

## Results

### Baseline characteristics and clinical parameters at the time of CRRT initiation

The baseline characteristics and clinical and laboratory parameters at baseline are shown in Tables 1 and 2. The mean patient age was 64.7 ± 15.8 years, and 165 patients (64.0%) were male. The primary cause of AKI was sepsis (*n* = 157, 60.9%) followed by ischemic injuries (*n* = 52, 20.2%). Among the total 258 patients, 157 patients (61%) died within 28 days after CRRT initiation. The patients were stratified into two groups, the 28-day survivors and the non-survivors. The SOFA, APACHE II score, and the need for mechanical ventilation were significantly higher in the 28-day non-survivors than in the survivors. Moreover, there was significantly more malignancy in the non-survivors compared with the survivors, while the survivors had significantly more hypertension compared with non-survivors (Table 1). Furthermore, the mean SBP and MAP were significantly higher in the survivors compared with the non-survivors. The mean platelet counts were significantly increased in the survivors compared with the non-survivors, whereas the mean total bilirubin level was significantly decreased in the survivors. There were no significant differences in the type of fluid administration or replacement of 20% albumin between the two groups, but the survivors received more enteral nutritional fluid than the non-survivors. The prescriptions for CRRT did not differ between two groups except for CRRT duration, which was longer in the survivors (Table 2). We further compared clinical parameters between the two groups at 24-h and 72-h after CRRT initiation, respectively (Additional file 1: Table S1 and S2). The SOFA and APACHE II scores, and need for mechanical ventilation were significantly higher in the non-survivors than the survivors at both 24-h and 72-h assessment after CRRT initiation. Furthermore, the mean MAP and platelet counts were lower, whereas white blood cell counts and total bilirubin levels were higher in the non-survivors compared to the survivors at both 24-h and 72-h assessment after CRRT initiation.

**Table 1:** Baseline characteristics

	Overall (*n* = 258)	28-day survival		*P*
		Survivor (*n* = 101)	Non-survivor (*n* = 157)	
Demographic data				
Age, years	64.7 ± 15.8	64.0 ± 17.3	65.1 ± 14.8	0.59
Male, *n* (%)	165 (64.0)	66 (65.3)	99 (63.1)	0.41
ICU admission body weight, kg	61.2 ± 12.2	61.7 ± 12.3	60.8 ± 12.3	0.59
ICU admission BMI, kg/m^2^	22.9 ± 12.2	23.2 ± 4.0	22.7 ± 3.8	0.27
Cause of AKI, *n* (%)				0.67
Septic	157 (60.9)	58 (57.4)	99 (63.1)	
Cardiogenic	28 (10.9)	13 (12.9)	15 (9.6)	
Ischemic	52 (20.2)	23 (22.8)	29 (18.5)	
Postoperative	7 (2.7)	1 (1.0)	6 (3.8)	
Drug induced	11 (4.3)	5 (5.0)	6 (3.8)	
Others	3 (1.2)	1 (1.0)	2 (1.3)	
Comorbidities, *n* (%)				
Hypertension	125 (48.4)	58 (57.4)	67 (42.7)	0.01
Diabetes	93 (36.0)	42 (41.6)	51 (32.5)	0.09
CVDs	81 (31.4)	32 (31.7)	49 (31.2)	0.52
Malignancy	23 (9.0)	4 (4.0)	19 (12.1)	0.02
Charlson Comorbidity Index	6.5 ± 2.4	6.4 ± 2.3	6.5 ± 2.5	0.59
SOFA score	11.6 ± 3.9	9.8 ± 3.8	12.8 ± 3.5	< 0.001
APACHE II score	26.0 ± 6.7	22.9 ± 6.6	28.1 ± 6.0	< 0.001
Glasgow Coma Score	6.3 ± 4.0	8.0 ± 4.3	5.2 ± 3.4	< 0.001
Mechanical ventilation needs, *n* (%)	203 (79.9)	69 (69.0)	134 (87.0)	< 0.001
FiO_2_	0.5 ± 0.2	0.4 ± 0.2	0.6 ± 0.2	0.004

Data were presented as mean ± standard deviation or number (%)

*Abbreviation*: *ICU* intensive care unit, *BMI* body mass index, *AKI* acute kidney injury, *CVD* cardiovascular disease, *SOFA* Sequential Organ Failure Assessment, *APACHE* Acute Physiology and Chronic Health Evaluation, *FiO2* fraction of inspired oxygen

**Table 2:** Clinical and laboratory parameters at baseline

	Overall (*n* = 258)	28-day survival		*P*
		Survivor (*n* = 101)	Non-survivor (*n* = 157)	
Time from ICU admission to CRRT start, day	4.4 ± 7.7	4.3 ± 7.6	4.4 ± 7.7	0.54
SBP, mmHg	114.1 ± 22.8	118.9 ± 24.0	111.0 ± 21.5	0.007
DBP, mmHg	65.8 ± 16.0	67.7 ± 15.7	64.6 ± 16.1	0.13
MAP, mmHg	81.9 ± 15.9	84.8 ± 15.6	80.1 ± 15.9	0.02
Vasopressor use, *n* (%)	179 (69.4)	55 (54.5)	124 (79.0)	< 0.001
Dose (NE, μg/kg/min)	0.59 [0.26–1.66]	0.50 [0.15–1.13]	0.65 [0.31–1.76]	0.06
Type of fluid administration, *n* (%)				0.98
0.9% sodium chloride	62 (25.7)	25 (27.2)	37 (24.8)	
0.45% sodium chloride	18 (7.5)	7 (7.6)	11 (7.4)	
Dextrose	113 (46.9)	42 (45.7)	71 (47.7)	
Plasma solution	25 (10.4)	6 (6.5)	19 (12.8)	
Lactate Ringer’s solution	23 (9.5)	12 (13.0)	11 (7.4)	
Replacement of 20% albumin, *n* (%)	156 (60.7)	56 (56.0)	100 (63.7)	0.16
Enteral nutritional fluid, *n* (%)	28 (10.9)	19 (19.0)	9 (5.8)	0.001
Prescriptions of CRRT				
Duration of CRRT, h	5.4 ± 6.4	7.2 ± 7.8	4.3 ± 4.9	< 0.001
Target clearance (mL/kg/h)	36.7 [34.2–40.9]	36.8 [34.1–41.5]	36.6 [34.2–39.9]	0.24
Dialysate flow rate, mL/h	1033.5 ± 164.5	1031.2 ± 181.9	1034.9 ± 152.8	0.86
Replacement flow rate, mL/h	1211.1 ± 389.9	1258.9 ± 422.9	1180.1 ± 380.8	0.12
Blood flow rate, mL/min	113.6 ± 33.0	116.5 ± 33.4	11.7 ± 32.8	0.25
Anticoagulation use, *n* (%)	159 (61.6)	(66 (65.3)	93 (59.2)	0.19
Laboratory findings				
Creatinine, mg/dL	1.4 ± 0.5	1.4 ± 0.5	1.4 ± 0.4	0.95
eGFR, mL/min/1.73m^2^	22.0 ± 16.5	21.3 ± 17.3	22.4 ± 16.1	0.62
White blood cells, *n*/μL	13.1 ± 10.4	11.8 ± 6.0	14.0 ± 12.4	0.10
Hemoglobin, g/dL	9.4 ± 2.2	9.3 ± 1.9	9.5 ± 2.3	0.59
Platelets, × 10^3^/μL	126.8 ± 87.9	141.9 ± 91.3	117.0 ± 84.5	0.03
PT-INR	2.0 ± 3.7	1.6 ± 0.6	2.3 ± 4.7	0.14
Total bilirubin, mg/dL	2.9 ± 5.2	1.7 ± 3.3	3.7 ± 6.0	0.007
Aspartate aminotransferase, IU/L	362.5 ± 66.5	481.7 ± 140.8	285.1 ± 60.3	0.15
Alanine aminotransferase, IU/L	152.7 ± 30.8	209.0 ± 71.3	115.9 ± 20.4	0.14
Lactic acid, mg/dL	58.6 ± 5.4	57.6 ± 9.5	59.2 ± 6.5	0.89

Data were presented as mean ± standard deviation, median [interquartile range] or number (%)

*Abbreviation: ICU* intensive care unit, *CRRT* continuous renal replacement therapy, *SBP* systolic blood pressure, *DBP* diastolic blood pressure, *MAP* mean arterial pressure, *NE* norepinephrine, *eGFR* estimated glomerular filtration rate, *PT-INR* prothrombin time-international normalized ratio

### Change of CFB and cumulative input and output in survivors and non-survivors

**Fig. 1:**
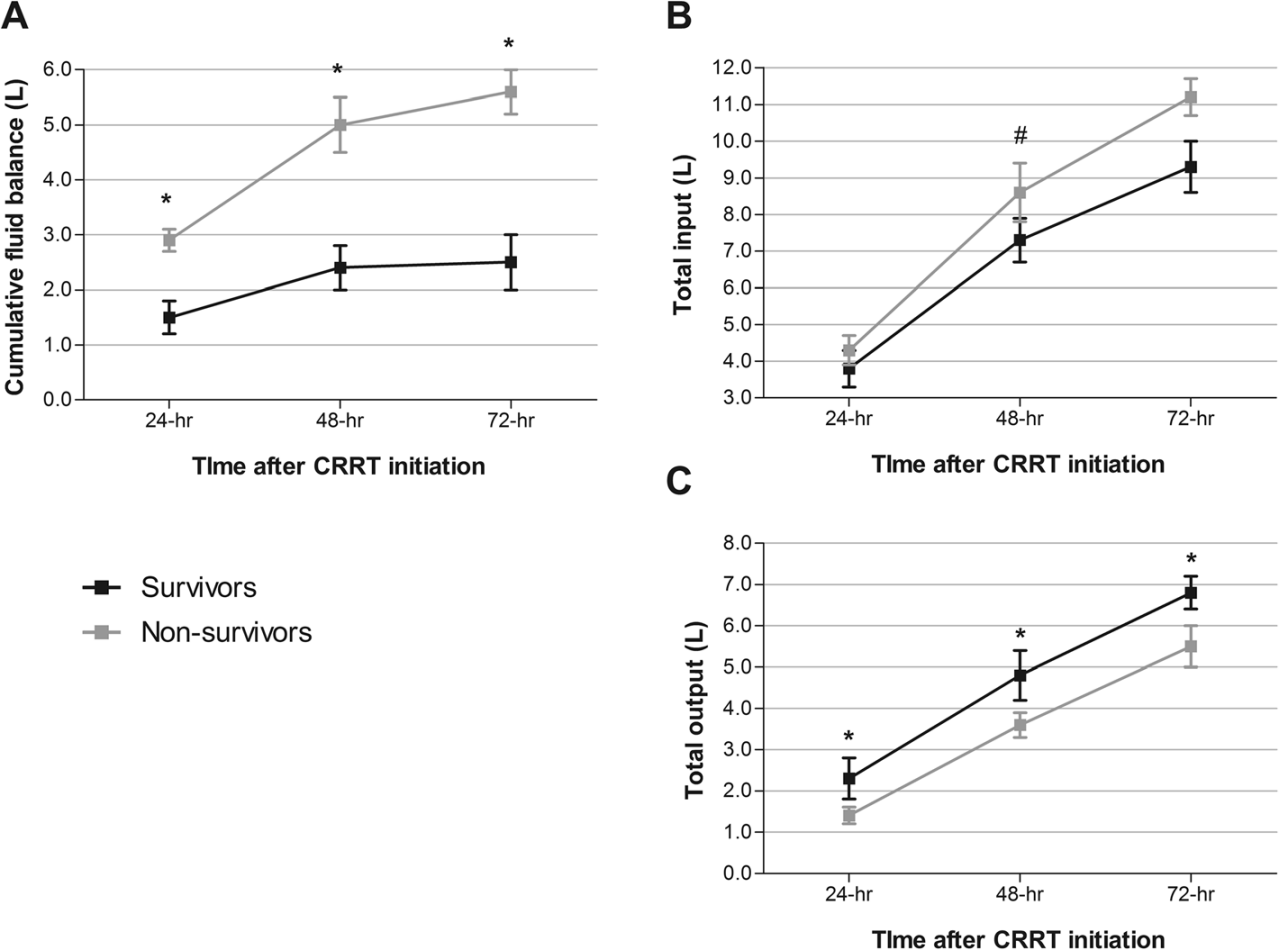
Comparison of fluid balance (**a**), total intake (**b**), and total output (**c**) between 28-day survivor vs. non-survivor. Each group was compared by two-way ANOVA, ^*^P < 0.001, ^#^*P* = 0.03. CRRT, continuous renal replacement therapy

We assessed the CFB and total cumulative input and output at 24-h and 72-h after initiation of CRRT, and Fig. 1 shows the change in each status. In the non-survivors, the CFBs at each time point were significantly higher than that in the survivors. In contrast, the total cumulative inputs at each time point were also higher in the non-survivors compared with that in the survivors, but a statistically significant difference was seen only at 48-h after CRRT start, while the total cumulative outputs at each time point were significantly lower in the non-survivors than those in the survivors.

### The effect of CFB on mortality risk

**Fig. 2:**
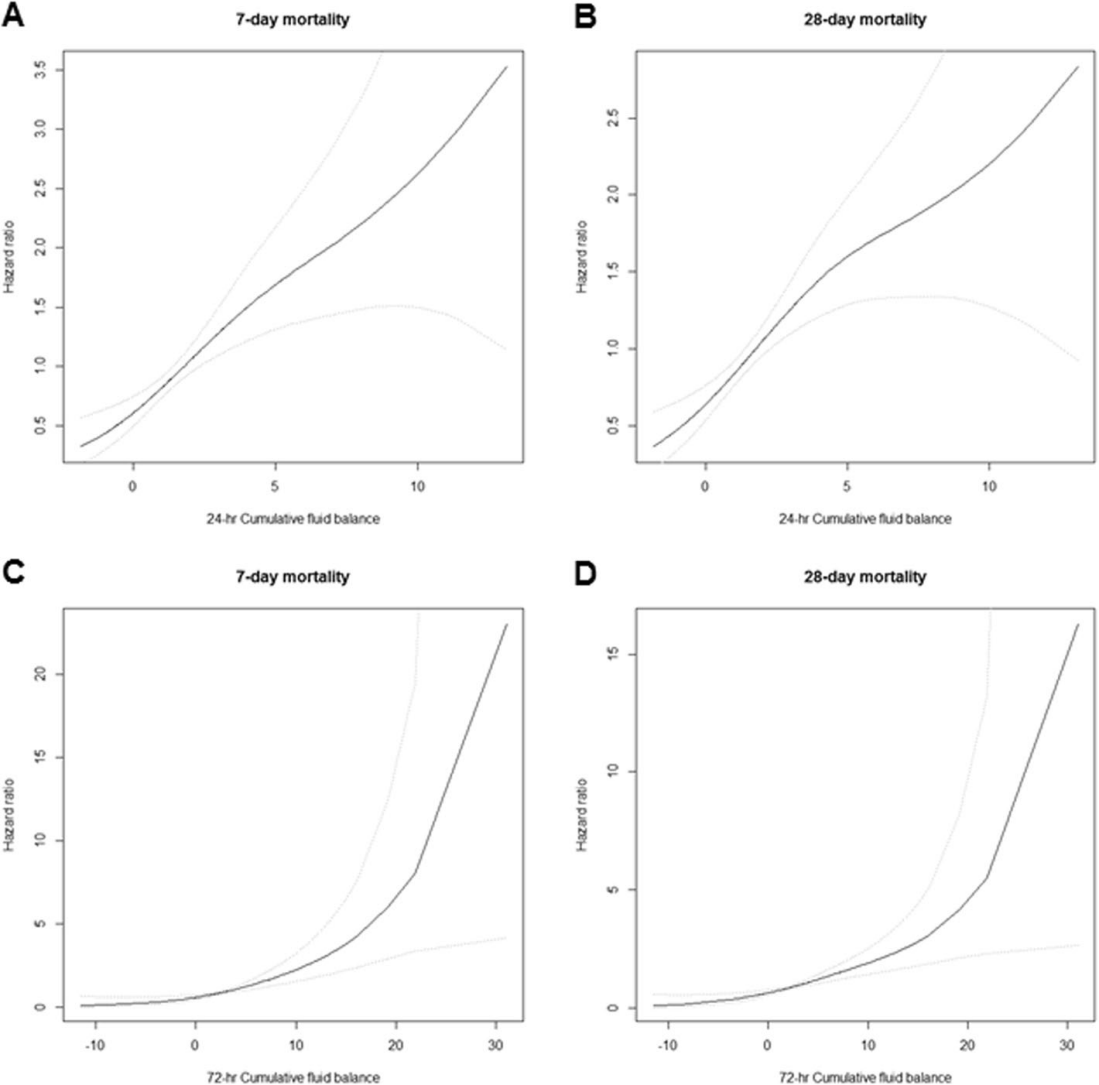
Cubic spline plots for the 7- or 28-day mortality risks according to 24-h (**a**, **b**) and 72-h (**c**, **d**) cumulative fluid balance. Black lines = hazard ratios, dotted lines = 95% confidence intervals

We next investigated the impact of 24- and 72-h CFB on 7- and 28-day mortality risk. Univariable Cox proportional hazard analysis showed that increased CFB at 24- and 72-h was significantly associated with an increase in 7-day mortality risk [increase of 1 L per 24-h CFB; hazard ratio (HR) = 1.15, 95% confidence interval (CI) = 1.09–1.22, and an increase of 1 L per 72-h CFB; HR = 1.12, 95% CI = 1.07–1.16] and 28-day mortality risk [increase of 1 L per 24-h CFB; HR = 1.14, 95% CI = 1.08–1.20 and increase of 1 L per 72-h CFB; HR = 1.10, 95% CI = 1.06–1.14]. Moreover, the increase of CFB at each time point was still significantly related to the elevation of 7-day mortality rate [increase of 1 L per 24-h CFB; HR = 1.14, 95% CI = 1.06–1.22 and increase of 1 L per 72-h CFB; HR = 1.10, 95% CI = 1.05–1.15] and 28-day mortality rate [increase of 1 L per 24-h CFB; HR = 1.11, 95% CI = 1.04–1.18 and increase of 1 L per 72-h CFB; HR = 1.07, 95% CI = 1.03–1.12], even after adjusting for confounding factors (Table 3 and Fig. 2).

**Table 3:** The association between cumulative fluid balance and mortality

	7-day mortality		28-day mortality	
	HR (95% CI)	*P*	HR (95% CI)	*P*
24-h cumulative fluid balance* (per 1.0 L increase, *n* = 258)				
Unadjusted	1.15 (1.09–1.22)	< 0.001	1.14 (1.08–1.20)	< 0.001
Adjusted model†	1.14 (1.06–1.22)	< 0.001	1.11 (1.04–1.18)	0.002
72-h cumulative fluid balance* (per 1.0 L increase, *n* = 191)				
Unadjusted	1.12 (1.07–1.16)	< 0.001	1.10 (1.06–1.14)	< 0.001
Adjusted model†	1.10 (1.05–1.15)	< 0.001	1.07 (1.03–1.12)	0.001

*Per 1.0 L increase

†Adjusted for age, sex, body mass index, mean arterial pressure, Charlson Comorbidity Index, history of diabetes and hypertension, hemoglobin, baseline estimated glomerular filtration rate, Sequential Organ Failure Assessment score, use of vasopressor, and type of fluid administration. Mean arterial pressure, hemoglobin, Sequential Organ Failure Assessment score, use of vasopressor use, and type of fluid administration at each of baseline and 72-h after CRRT initiation were used for adjustment in 24-h and 72-h CFB models, respectively

*Abbreviation: HR* hazard ratio, *CI* confidence interval

### The interactive effect of cumulative input and output on the impact of CFB on mortality

**Fig. 3:**
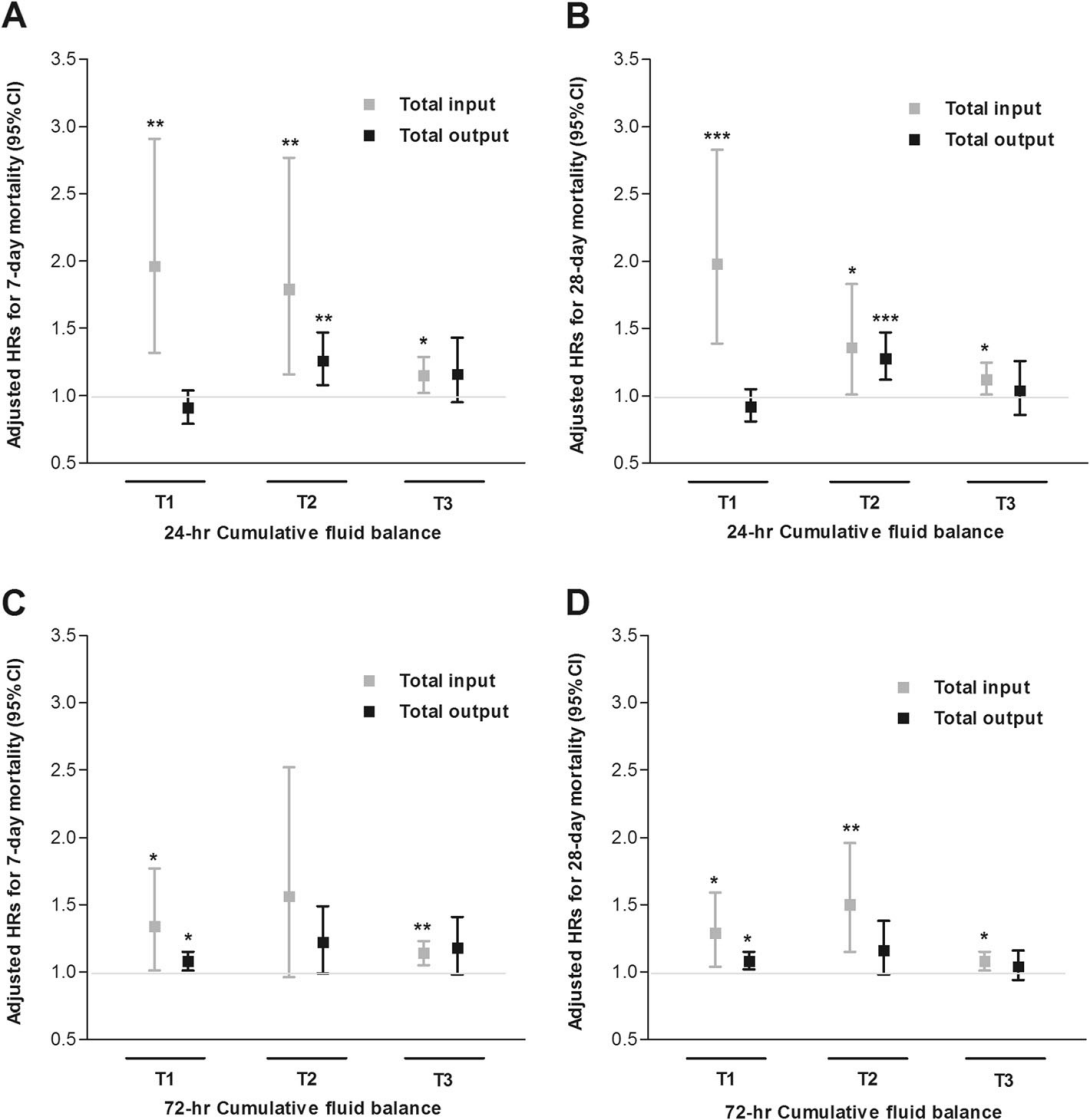
Comparison of adjusted hazard ratios for 7-day or 28-day mortality according to 24-h (**a**, **b**) or 72-h (**c**, **d**) cumulative fluid balance stratified with tertiles of total input and output. T1–3 represents tertile groups of total input and output. The exact values of T1–3 are presented in Table 4. Models were adjusted for age, sex, body mass index, mean arterial pressure, Charlson comorbidity index, history of diabetes and hypertension, hemoglobin, baseline estimated glomerular filtration rate, Sequential Organ Failure Assessment score, use of vasopressor, and type of fluid administration. Mean arterial pressure, hemoglobin, Sequential Organ Failure Assessment score, use of vasopressor use, and type of fluid administration at each of baseline and 72-h after CRRT initiation were used for adjustment in 24-h and 72 h-cumulative fluid balance models, respectively. ^*^P < 0.05, ^**^P < 0.01, ^***^P < 0.001. CRRT, continuous renal replacement therapy

We stratified the patients into three groups based on the tertile of 24-h and 72-h cumulative input or output. The baseline characteristics of each group are shown in Additional file 1: Table S3. The patients in the higher input tertile were younger and showed higher baseline eGFR levels and SOFA scores than those in the lowest tertile at both 24-h and 72-h assessment after CRRT initiation. However, output tertile groups showed no significant differences in clinical characteristics, including blood pressure, comorbidity status, and SOFA scores at either 24-h or 72-h assessment after CRRT initiation. We then investigated the impact of CFB on the mortality in each group. Increases in 24-h CFB were significantly associated with the increase in 7- and 28-day mortality risks irrespective of the cumulative input, even after adjusting for age, sex, BMI, MAP, CCI, history of diabetes and hypertension, hemoglobin, eGFR, SOFA score, use of vasopressors, and type of fluid administration (Table 4 and Fig. 3). Moreover, the increases in 72-h CFB were also significantly related to the increase in 7- and 28-day mortality risk regardless of cumulative input, except for the T2 group for 7-day mortality. However, we did not find significant associations between the increase in 24- and 72-h CFB and 7- and 28-day mortality risk in the groups stratified according to cumulative output tertile. Instead, CFB was significantly related to an increase in 7- and 28-day mortality risk only in the T2 group of the 24-h cumulative output, and the T1 group of the 72-h cumulative output. Taken together, we surmise that the impact of CFB on mortality might be dependent on cumulative output (Table 4).

**Table 4:** The association between 24-h and 72-h cumulative fluid balance and mortality at different levels of total input and output

	7-day mortality		28-day mortality	
	HR (95% CI)	*P*	HR (95% CI)	*P*
	24-h cumulative fluid balance* (per 1.0 L increase, *n* = 258)†			
Total input (L)				
T1 (≤ 2.81)	1.96 (1.32–2.91)	0.001	1.98 (1.39–2.83)	< 0.001
T2 (2.82–4.36)	1.79 (1.16–2.77)	0.008	1.36 (1.01–1.83)	0.04
T3 (> 4.36)	1.15 (1.02–1.29)	0.02	1.12 (1.01–1.25)	0.04
Total output (L)				
T1 (≤ 0.69)	0.91 (0.79–1.04)	0.18	0.92 (0.81–1.05)	0.22
T2 (0.70–2.13)	1.26 (1.08–1.47)	0.003	1.28 (1.12–1.47)	< 0.001
T3 (> 2.13)	1.16 (0.95–1.43)	0.15	1.04 (0.86–1.26)	0.70
	72-h cumulative fluid balance* (per 1.0 L increase, *n* = 191)†			
Total input (L)				
T1 (≤ 7.48)	1.34 (1.01–1.77)	0.04	1.29 (1.04–1.59)	0.02
T2 (7.49–11.1)	1.56 (0.96–2.52)	0.07	1.50 (1.15–1.96)	0.003
T3 (> 11.1)	1.14 (1.05–1.23)	0.001	1.08 (1.01–1.15)	0.02
Total output (L)				
T1 (≤ 4.27)	1.08 (1.01–1.15)	0.03	1.08 (1.02–1.15)	0.01
T2 (4.28–7.14)	1.22 (0.99–1.49)	0.06	1.16 (0.98–1.38)	0.09
T3 (> 7.14)	1.18 (0.98–1.41)	0.08	1.04 (0.94–1.16)	0.44

*per 1.0 L increase

†Adjusted for age, sex, body mass index, mean arterial pressure, Charlson Comorbidity Index, history of diabetes and hypertension, hemoglobin, baseline estimated glomerular filtration rate, Sequential Organ Failure Assessment score, use of vasopressor, and type of fluid administration. Mean arterial pressure, hemoglobin, Sequential Organ Failure Assessment score, use of vasopressor use, and type of fluid administration at each of baseline and 72-h after CRRT initiation were used for adjustment in 24-h and 72 h-CFB models, respectively

*Abbreviation: HR* hazard ratio, *CI* confidence interval, *T* tertiles

## Discussion

This study shows the effects of cumulative input and output on the impact of short-term (24-h) and relatively long-term (72-h) CFB on 7- and 28-day mortality risk among AKI patients undergoing CRRT. We found that both increases in short-term and longer-term CFB were significantly associated with increases in 7- and 28-day mortality risk, and the effect of CFB on mortality might be dependent on cumulative output.

The main concern in AKI patients undergoing CRRT is how to control their volume status [26, 27]. Several studies have examined this issue, and almost all suggested that fluid overload was significantly associated with increased mortality risk as well as aggravation of kidney function [2, 16, 28]. Although most studies have focused on a negative fluid balance in AKI patients with CRRT, physicians need to consider volume resuscitation, since it is crucial to restore and maintain hemodynamic stability in critically ill patients [29–32]. To reduce CFB, physicians need to choose low fluid resuscitation and high removal of resuscitation as much as possible. We stratified the patients in this study into three groups based on 24-h and 72-h cumulative input or output tertiles to reveal the interactive effect of cumulative input or output on the impact of CFB on mortality. We found a big VIF between CFB and cumulative input/output, respectively, and it was difficult to adjust cumulative input and output to show the impact of CFB on mortality risk, independently. We also found that the increase in CFB was still significantly associated with an increase in mortality risk irrespective of the stratification of cumulative input, which suggests that removal of more fluid output may reduce the mortality risk. In contrast, when we performed Cox analysis for mortality after patients were stratified based on cumulative output, there was no significant association between CFB increase and mortality risk, which means that reduction of cumulative input might not decrease mortality risk.

Moreover, we also investigated the characteristics of the three patient groups stratified by their cumulative input and output at 24-h and 72-h after CRRT initiation (Additional file 1: Table S3). Especially, there were no significant differences in MAP, CCI, and SOFA scores among the three groups stratified by output amount, suggesting that fluid removal might be done irrespective of illness severity. Therefore, we do not think a higher rate of fluid removal is simply a marker of lower illness severity. Instead, more fluid removal could provide an indirect benefit against the oxygen-mismatched diffusion and distorted tissue architecture that originate from fluid overload and tissue edema. Taken together, we suggest that physicians need to pay closer attention to decreasing CFB, especially by increasing fluid removal, to improve the clinical outcomes of their AKI patients receiving CRRT.

Adequate fluid resuscitation is essential to restore tissue perfusion in critically ill patients with AKI [27, 33, 34], and fluid administration contributes to improve glomerular filtration [9]. Moreover, fluid administration is aimed to restore systemic blood pressure, a major determinant of renal perfusion pressure and cardiac output [35–37]. In particular, patients with severe sepsis need adequate fluid resuscitation within 1 to 3 h of disease onset to overcome their hemodynamically unstable state [38, 39]. Thus, clinicians may try to administer more fluid to restore tissue perfusion and to improve disease course. However, fluid overload and tissue edema physiologically result in impaired diffusion of oxygen and metabolites, distorted tissue architecture, obstruction of capillary blood flow or lymphatic drainage, and disturbed cell-cell interactions. These processes subsequently contribute to progressive organ dysfunction. The patients in the current study were more likely to be overhydrated at CRRT start, and their severity scores (SOFA and APACHE II score) were higher than those in other studies [25, 40]. Thus, more removal of patient output seems to be more effective for a decrease in mortality compared with lower cumulative input, and interpretation of the current study should be made with caution. Generally, most AKI patients who require CRRT are critically ill and in an overhydrated condition [27, 41]. Moreover, effective circulating volume appears to be low in these patients [8]. Thus, we surmise that it might be more helpful to increase fluid removal than to decrease input. Furthermore, Murugan et al. [42] recently showed that higher ultrafiltration intensity (> 25 mL/kg/day) was associated with lower risk for mortality compared with lower ultrafiltration intensity (≤ 20 mL/kg/day) among critically ill patients with fluid overload who were undergoing CRRT. The authors suggested several reasons for the benefit of intensive removal. First, increase in cumulative output may reduce the risk of subsequent edema-related organ dysfunction [43]. Second, the increase in cumulative output by intensive net ultrafiltration may lead to better clearance of unknown molecules and contribute to better survival independently of fluid balance [44].

There are several limitations in the current study. First, this study was a retrospective study conducted in a single center with a relatively small sample size. Thus, selection bias was not completely avoidable, and these results may not be applicable to other ethnicities. Nevertheless, we assessed the power of our study sample size, and it was more than 80%, showing the robustness of our study sample size (Additional file 1: Table S4). In addition, previous studies with AKI patients undergoing CRRT were performed with sample sizes similar to ours [3, 45]. Moreover, this is the first study, to the best our knowledge, to reveal the interactive effect of fluid input and output in managing fluid balance for CRRT-treated AKI patients, so it can serve as precedent research for future prospective interventional studies with more patients that are needed to verify our study results. Second, we could not measure the effects of diet or drugs used or insensible water loss. However, most of these patients could not eat and nutritional support was provided via an infused solution for 72-h from CRRT start [only 28 (10.9%) patients were supplied with enteral nutritional fluid]. Third, physicians wanted to increase mechanical fluid removal when their patients could endure the increase in fluid removal, suggesting that patients whose removal was increased would experience better clinical outcomes. Several studies investigating the effect of fluid overload on mortality in critically ill patients have been performed, and most of these studies showed that a high CFB was significantly associated with increased mortality risk. However, it seems to be a case of the chicken and the egg. It can be difficult to increase fluid removal when patients are hemodynamically unstable, which suggests that greater illness severity, rather than increased CFB might be leading to worse clinical outcomes. In this study, however, we tried to adjust the severity indices, such as MAP, CCI, and SOFA score, to exclude the effect of those factors on mortality and we found that high CFB was still significantly related to an increase in 7- and 28-day mortality independently of the severity indices. Taken together, we surmise that fluid removal itself might provide an indirect benefit for clinical outcomes by improving tissue oxygenation, not just that a higher rate of fluid removal is a marker of lower severity of illness. Fourth, we arbitrarily stratified these patients into three groups based on cumulative input and/or output to examine the interaction of input and/or output on CFB impact on mortality. However, we investigated the interaction between CFB and cumulative input and output and found that VIF was too large to be adjusted with CFB. Thus, we decided to analyze the effect of cumulative input and output on the impact of CFB on mortality after stratifying the patients. Finally, we used eGFR as a marker of the patients’ renal function status, even though it is not a valid reflection of renal function in an acute setting. In this study, we could not have evaluated baseline kidney function with gold standard methods for GFR measurement such as inulin clearance, so we only used calculated eGFR values with MDRD equation. Further study is needed to accurately compare baseline kidney function measured by gold standard methods. Despite these limitations, this study reports robust clinical findings for the effect of an increase in short-term and longer-term CFB on mortality risk and the dependence of CFB on cumulative output.

## Conclusions

In conclusion, an increase in CFB was significantly associated with an increase in mortality risk in AKI patients undergoing CRRT, irrespective of cumulative period. Moreover, the impact of CFB on mortality might be dependent on cumulative output. Although a prospective interventional study with a larger number of patients will be needed, this study revealed that physicians need to decrease the CFB of CRRT patients as much as possible and to consider increasing the amount of fluid removal.

## Supplementary information

**Additional file 1:** **Figure S1.** Flow diagram for patient enrollment. **Table S1.** Clinical parameters at 24-hr after initiation of continuous renal replacement therapy. **Table S2.** Clinical parameters at 72-hr after initiation of continuous renal replacement therapy. **Table S3.** Comparison of patient’s characteristics according to amount of input or output at 24-hr and 72-hr assessment after CRRT initiation. **Table S4.** Power analysis to assess validity of the study sample size.

## Data Availability

The data that support the findings of this study are available from the corresponding author upon reasonable request.

## Abbreviations

AKI: Acute kidney injury
APACHE: Acute Physiology and Chronic Health Evaluation
BMI: Body mass index
CCI: Charlson Comorbidity index
CFB: Cumulative fluid balance
CRRT: Continuous renal replacement therapy
DBP: Diastolic blood pressure
eGFR: Estimated glomerular filtration rate
ESRD: End-stage renal disease
FiO_2_: Fraction of inspired oxygen
GCS: Glasgow Coma Score
ICU: Intensive care unit
IRB: Institutional Review Board
MAP: Mean arterial pressure
SBP: Systolic blood pressure
SOFA: Sequential Organ Failure Assessment
VIF: Variance inflation factor
