# Supplementary material for: The interactive effects of input and output on managing fluid balance in patients with acute kidney injury requiring continuous renal replacement therapy
Source: Crit Care. 2019 Oct 29;23:329. doi: 10.1186/s13054-019-2633-0 (PMC6819592; doi:10.1186/s13054-019-2633-0)
Supplement: Supplementary file 1 — Additional file 1: Figure S1. Flow diagram for patient enrollment. Table S1. Clinical parameters at 24-hr after initiation of continuous renal replacement therapy. Table S2. Clinical parameters at 72-hr after initiation of continuous renal replacement therapy. Table S3. Comparison of patient’s characteristics according to amount of input or output at 24-hr and 72-hr assessment after CRRT initiation. Table S4. Power analysis to assess validity of the study sample size. [file 13054_2019_2633_MOESM1_ESM.docx]

**ADDITIONALFILE**
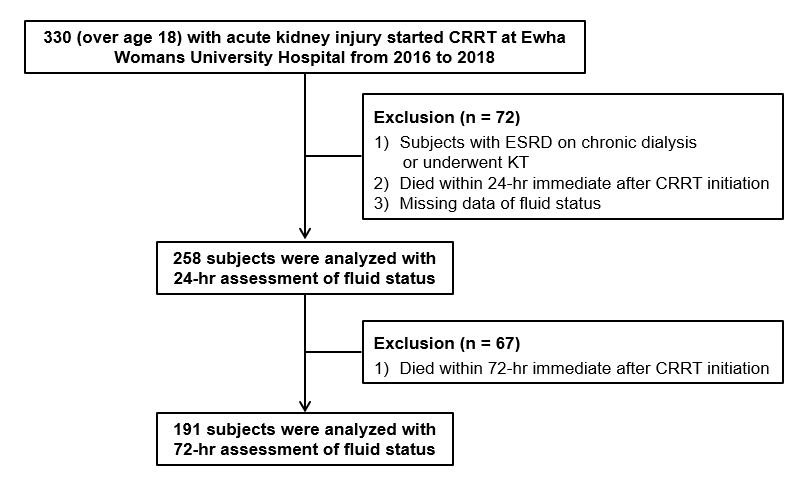


**Figure S1.** Flow diagram for patient enrollment.

***Abbreviation:*** CRRT, continuous renal replacement therapy; ESRD, end stage renal disease; KT, kidney transplantation

**Table S1.** Clinical parameters at 24-hr after initiation of continuous renal replacement therapy

|  |  | **28-day Survival** | | |  |
| --- | --- | --- | --- | --- | --- |
|  | **Overall**  **(*n* = 226)** | | **Survivor**  **(*n* = 101)** | **Non-survivor**  **(*n* = 125)** | ***P*** |
| **SOFA score** | 11.9 ± 4.1 | | 9.5 ± 3.6 | 13.6 ± 3.5 | <0.001 |
| **APACHE II score** | 23.0 ± 6.9 | | 19.7 ± 5.7 | 25.4 ± 6.8 | <0.001 |
| **Glasgow Coma Score** | 5.5 ± 4.4 | | 7.5 ± 4.7 | 4.1 ± 3.5 | <0.001 |
| **Mechanical ventilation needs, n (%)** | 203 (79.9) | | 69 (69.0) | 134 (87.0) | <0.001 |
| FiO2 | 0.5 ± 0.2 | | 0.4 ± 0.1 | 0.6 ± 0.2 | <0.001 |
| **SBP, mmHg** | 118.1 ± 23.9 | | 125.9 ± 22.3 | 112.4 ± 23.5 | <0.001 |
| **DBP, mmHg** | 66.8 ± 16.9 | | 70.0 ± 16.8 | 64.4 ± 16.8 | 0.01 |
| **MAP, mmHg** | 78.0 ± 26.9 | | 83.4 ± 25.9 | 74.2 ± 27.1 | 0.001 |
| **Vasopressor dose (NE, mcg/kg/min)** | 2.6 ± 0.2 | | 3.3 ± 0.5 | 2.4 ± 0.2 | 0.88 |
| **Type of fluid administration, n (%)** |  | |  |  | 0.98 |
| 0.9% sodium chloride | 62 (25.7) | | 25 (27.2) | 37 (24.8) |  |
| 0.45% sodium chloride | 18 (7.5) | | 7 (7.6) | 11 (7.4) |  |
| Dextrose | 113 (46.9) | | 42 (45.7) | 71 (47.7) |  |
| Plasma solution | 25 (10.4) | | 6 (6.5) | 19 (12.8) |  |
| Lactate Ringer’s solution | 23 (9.5) | | 12 (13.0) | 11 (7.4) |  |
| **Replacement of 20% albumin, n (%)** | 156 (60.7) | | 56 (56.0) | 100 (63.7) | 0.16 |
| **Enteral nutritional fluid, n (%)** | 28 (10.9) | | 19 (19.0) | 9 (5.8) | 0.001 |
| **Prescriptions of CRRT** |  | |  |  |  |
| Target clearance (mL/kg/h) | 22.4 ± 4.7 | | 22.8 ± 4.6 | 22.2 ± 4.9 | 0.33 |
| **Laboratory findings** |  | |  |  |  |
| Creatinine, mg/dL | 2.3 ± 1.7 | | 2.3 ± 1.2 | 2.3 ± 1.1 | 0.58 |
| White blood cells, n/μL | 12.3 ± 9.3 | | 10.8 ± 5.4 | 13.4 ± 11.2 | 0.04 |
| Hemoglobin, g/dL | 8.9 ± 1.6 | | 8.8 ± 1.5 | 9.1 ± 1.7 | 0.19 |
| Platelets, x10^3^/μL | 92.3 ± 63.3 | | 106.5 ± 68.8 | 81.9 ± 57.1 | 0.004 |
| PT-INR | 2.1 ± 3.1 | | 1.6 ± 0.5 | 2.5 ± 3.9 | 0.04 |
| Total bilirubin, mg/dL | 3.4 ± 5.2 | | 2.0 ± 3.1 | 4.4 ± 6.1 | 0.001 |
| Aspartate aminotransferase, IU/L | 1489.5 ± 4610.9 | | 977.4 ± 2951.9 | 1863.8 ± 5498.5 | 0.15 |
| Alanine aminotransferase, IU/L | 413.8 ± 1061.1 | | 323.3 ± 838.6 | 478.7 ± 1194.4 | 0.28 |
| Lactic acid, mg/dL | 44.9 ± 48.2 | | 24.3 ± 22.1 | 60.7 ± 56.3 | 0.12 |
| Data were presented as mean ± standard deviation, median [interquartile range] or number (%)  ***Abbreviation:*** ICU, intensive care unit; CRRT, continuous renal replacement therapy; SBP, systolic blood pressure; DBP, diastolic blood pressure; MAP, mean arterial pressure; NE, norepinephrine; eGFR, estimated glomerular filtration rate; PT-INR, prothrombin time-international normalized ratio | | | | | |

**Table S2.** Clinical parameters at 72-hr after initiation of continuous renal replacement therapy

|  |  | **28-day Survival** | | |  |
| --- | --- | --- | --- | --- | --- |
|  | **Overall**  **(*n* = 191)** | | **Survivor**  **(*n* = 101)** | **Non-survivor**  **(*n* = 90)** | ***P*** |
| **SOFA score** | 11.1 ± 4.1 | | 8.9 ± 3.2 | 13.6 ± 3.4 | <0.001 |
| **APACHE II score** | 29.6 ± 6.6 | | 16.7 ± 5.4 | 23.1 ± 6.1 | <0.001 |
| **Glasgow Coma Score** | 3.7 ± 4.5 | | 6.4 ± 5.1 | 1.9 ± 3.1 | <0.001 |
| **Mechanical ventilation needs, n (%)** | 113 (80.1) | | 52 (68.4) | 61 (93.8) | <0.001 |
| FiO2 | 0.5 ± 0.2 | | 0.4 ± 0.1 | 0.6 ± 0.2 | <0.001 |
| **SBP, mmHg** | 121.1 ± 22.3 | | 123.6 ± 21.3 | 118.2 ± 23.4 | 0.16 |
| **DBP, mmHg** | 67.4 ± 14.1 | | 69.5 ± 14.3 | 64.9 ± 13.6 | 0.05 |
| **MAP, mmHg** | 55.8 ± 42.2 | | 74.1 ± 34.1 | 42.6 ± 42.8 | <0.001 |
| **Vasopressor dose (NE, mcg/kg/min)** | 2.2 ± 2.7 | | 2.4 ± 2.8 | 2.1 ± 2.7 | 0.72 |
| **Type of fluid administration, n (%)** |  | |  |  | 0.82 |
| 0.9% sodium chloride | 36 (34.0) | | 17 (32.1) | 19 (35.8) |  |
| 0.45% sodium chloride | 18 (17.0) | | 10 (18.9) | 8 (15.1) |  |
| Dextrose | 28 (26.4) | | 14 (26.4) | 14 (26.4) |  |
| Plasma solution | 14 (13.2) | | 6 (11.3) | 8 (15.1) |  |
| Lactate Ringer’s solution | 10 (9.4) | | 6 (11.3) | 4 (7.5) |  |
| **Replacement of 20% albumin, n (%)** | 142 (74.3) | | 70 (69.3) | 72 (80.0) | 0.30 |
| **Enteral nutritional fluid, n (%)** | 43 (22.5) | | 30 (29.7) | 13 (14.4) | 0.01 |
| **Prescriptions of CRRT** |  | |  |  |  |
| Target clearance (mL/kg/h) | 23.3 ± 4.8 | | 23.4 ± 4.7 | 23.3 ± 5.0 | 0.96 |
| **Laboratory findings** |  | |  |  |  |
| Creatinine, mg/dL | 1.5 ± 0.7 | | 1.5 ± 0.7 | 1.5 ± 0.6 | 0.80 |
| White blood cells, n/μL | 12.5 ± 8.4 | | 10.7 ± 6.1 | 14.6 ± 10.1 | 0.005 |
| Hemoglobin, g/dL | 8.9 ± 1.4 | | 8.8 ± 1.3 | 9.1 ± 1.5 | 0.23 |
| Platelets, x10^3^/μL | 86.2 ± 6.3 | | 100.2 ± 75.2 | 69.5 ± 51.5 | 0.01 |
| PT-INR | 2.3 ± 6.9 | | 2.7 ± 9.4 | 1.8 ± 0.8 | 0.50 |
| Total bilirubin, mg/dL | 4.8 ± 6.3 | | 2.9 ± 3.9 | 7.3 ± 7.8 | <0.001 |
| Aspartate aminotransferase, IU/L | 386.2 ± 830.4 | | 392.3 ± 741.2 | 378.9 ± 931.0 | 0.92 |
| Alanine aminotransferase, IU/L | 215.8 ± 450.3 | | 258.3 ± 496.5 | 165.4 ± 386.4 | 0.22 |
| Lactic acid, mg/dL | 18.7 ± 19.9 | | 14.8 ± 19.0 | 23.2 ± 20.4 | 0.12 |
| Data were presented as mean ± standard deviation, median [interquartile range] or number (%)  ***Abbreviation:*** ICU, intensive care unit; CRRT, continuous renal replacement therapy; SBP, systolic blood pressure; DBP, diastolic blood pressure; MAP, mean arterial pressure; NE, norepinephrine; eGFR, estimated glomerular filtration rate; PT-INR, prothrombin time-international normalized ratio | | | | | |

**Table S3.** Comparison of patient’s characteristics according to amount of input or output at 24-hr and 72-hr assessment after CRRT initiation

|  | **24-hr assessment** | | | | **72-hr assessment** | | | |
| --- | --- | --- | --- | --- | --- | --- | --- | --- |
|  | **T1** | **T2** | **T3** | ***P*** | **T1** | **T2** | **T3** | ***P*** |
| **Input** |  |  |  |  |  |  |  |  |
| Age, years | 68.7 ± 13.3 | 63.3 ± 17.1 | 61.9 ± 16.1 | 0.01 | 68.1 ± 15.2 | 64.4 ± 17.1 | 60.4 ± 15.6 | 0.03 |
| MAP, mmHg | 82.0 ± 14.7 | 82.3 ± 15.8 | 81.3 ± 17.4 | 0.92 | 84.2 ± 15.9 | 84.3 ± 14.7 | 80.9 ± 16.9 | 0.39 |
| Charlson comorbidity index | 6.7 ± 2.5 | 6.5 ± 2.2 | 6.2 ± 2.5 | 0.54 | 7.2 ± 2.4 | 6.2 ± 2.2 | 6.1 ± 2.4 | 0.03 |
| eGFR at CRRT initiation, (mL/kg/1.73m^2^) | 19.5 ± 16.2 | 19.7 ± 13.6 | 26.6 ± 18.5 | 0.01 | 18.1 ± 14.5 | 19.3 ± 14.6 | 26.3 ± 19.5 | 0.01 |
| SOFA score | 10.5 ± 3.8 | 11.5 ± 4.2 | 12.9 ± 3.3 | <0.001 | 9.2 ± 3.6 | 11.0 ± 4.1 | 12.8 ± 3.4 | <0.001 |
| **Output** |  |  |  |  |  |  |  |  |
| Age, years | 66.6 ± 14.3 | 67.7 ± 13.9 | 60.6 ± 18.1 | 0.01 | 66.5 ± 15.2 | 66.2 ± 15.2 | 60.2 ± 17.7 | 0.04 |
| MAP, mmHg | 79.6 ± 15.7 | 81.5 ± 16.2 | 84.4 ± 15.8 | 0.14 | 81.9 ± 16.8 | 83.9 ± 15.7 | 83.7 ± 15.1 | 0.74 |
| Charlson comorbidity index | 6.2 ± 2.4 | 6.9 ± 2.4 | 6.3 ± 2.4 | 0.12 | 6.8 ± 2.3 | 6.6 ± 2.2 | 6.2 ± 2.6 | 0.29 |
| eGFR at CRRT initiation, (mL/kg/1.73m^2^) | 21.8 ± 16.7 | 20.3 ± 15.2 | 23.9 ± 17.6 | 0.34 | 18.6 ± 15.5 | 20.3 ± 15.1 | 24.9 ± 18.8 | 0.10 |
| SOFA score | 12.2 ± 4.0 | 10.9 ± 3.7 | 11.6 ± 3.8 | 0.09 | 10.2 ± 3.7 | 10.9 ± 4.2 | 11.8 ± 3.9 | 0.08 |
| Data were presented as mean ± standard deviation, median [interquartile range] or number (%)  ***Abbreviation:*** SOFA, Sequential Organ Failure Assessment | | | | | | | | |

**Table S4.** Power analysis to assess validity of the study sample size

|  | **Sample size** | **Regression coefficient** | **Standard deviation** | **Event**  **rate** | **R^2^** | **Two-sided alpha** | **Beta** | ***Power*** |
| --- | --- | --- | --- | --- | --- | --- | --- | --- |
| **24-hr assessment** |  |  |  |  |  |  |  |  |
| 7-day mortality | 258 | 0.127 | 2.600 | 0.461 | 0.150 | 0.050 | 0.087 | 0.913 |
| 28-day mortality | 258 | 0.103 | 2.600 | 0.616 | 0.160 | 0.050 | 0.128 | 0.872 |
| **72-hr assessment** |  |  |  |  |  |  |  |  |
| 7-day mortality | 191 | 0.091 | 5.200 | 0.282 | 0.160 | 0.050 | 0.111 | 0.889 |
| 28-day mortality | 191 | 0.071 | 5.200 | 0.488 | 0.130 | 0.050 | 0.086 | 0.914 |
| ***Note:*** Power is the probability of rejecting a false null hypothesis. It should be close to one.  R^2^ is the R-squared achieved when cumulative fluid balance is regressed on the other covariates. Alpha is the probability of rejecting a true null hypothesis. Beta is the probability of accepting a false null hypothesis. PASS 13 Power Analysis and Sample Size Software was used for the analysis (NCSS, LLC. Kaysville, Utah, USA, ncss.com/software/pass. 2014). | | | | | | | | |
